# Lasso peptide MccY alleviates non-typhoidal salmonellae-induced mouse gut inflammation via regulation of intestinal barrier function and gut microbiota

**DOI:** 10.1128/spectrum.01784-23

**Published:** 2023-10-11

**Authors:** Yu Li, Wenjing Li, Zhiwei Zeng, Yu Han, Qinxi Chen, Xinyi Dong, Zepeng Wang, Saixiang Feng, Weisheng Cao

**Affiliations:** 1 College of Veterinary Medicine, South China Agricultural University, Guangzhou, China; 2 Key Laboratory of Zoonosis Prevention and Control of Guangdong Province, Guangdong, China; 3 Key Laboratory of Zoonosis of Ministry of Agriculture and Rural Affairs, Guangzhou, China; 4 Key Laboratory of Veterinary Vaccine Innovation of the Ministry of Agriculture and Rural Affairs, Guangzhou, China; 5 National and Regional Joint Engineering Laboratory for Medicament of Zoonosis Prevention and Control, Guangzhou, China; University of California, San Diego, La Jolla, California, USA

**Keywords:** lasso peptide, MccY, Non-Typhoid *Salmonella*, intestinal epithelial barrier, gut dysbiosis

## Abstract

Non-typhoidal salmonellae-induced zoonotic intestinal bleeding infections are a crucial threat to public health worldwide, and the development of new medications is required to prevent and treat non-typhoid *Salmonella* (NTS) infections. MccY, a class of lasso peptide, is effective in biological sterilization against *Salmonella* and *Shigella*. In this study, the therapeutic effect of MccY on *S*. Typhimurium-infected BALB/c mice has been discussed. specific pathogen free (SPF) mice were infected with the ST53 strain with bacterial loads of 10^6^ and 10^3^ CFU/mL, respectively, and then treated with 9.92 mg/kg BW MccY. Results revealed that MccY significantly reduced mouse mortality by 20%–40% and effectively alleviated weight loss and diarrhea. The integrity of the intestinal structure was protected, and the expression of TNF-α, IL-6, IL-10, and IL-18 was inhibited. Particularly, MccY exerted multiple effects on gut microbiota ecology, including direct elimination of *Salmonella*, showing promise in limiting infection to the gut level and thereby halting the transmission of NTS. MccY indirectly inhibited other opportunistic pathogens and partially restored the microbiota, promoting an increase in *Bacteroidetes*, *Lactobacillus*, *Muribaculum*, and *Clostridium*. Consequently, this work provides an understanding of the link between host infection and therapeutic application of MccY.

**IMPORTANCE:**

Diseases caused by *Enterobacteriaceae* multidrug-resistant strains have become increasingly difficult to manage. It is necessary to verify the new antibacterial drug MccY effect on non-typhoid *Salmonella* infection in mice since it is regarded as a promising microcin. The results demonstrated that MccY has a potential therapeutic application value in the protection against *Salmonella*-induced intestinal damage and alleviating related intestinal dysbiosis and metabolic disorders. MccY could be a promising candidate as an antimicrobial or anti-inflammatory agent for treating infectious diseases.

## INTRODUCTION

The expansion of enterobacteria is a hallmark of microbial imbalance known as “dysbiosis.” Diseases caused by *Enterobacteriaceae* multidrug-resistant strains have become increasingly difficult to manage, including *Escherichia coli, Salmonella*. and *Shigella* (1). *Salmonella* is crucial foodborne pathogen that causes diarrheal disease, intestinal dysbiosis, host intestinal barrier damage, and mortality in young animals and humans (2). An estimated 800,000 to 3.7 million cases of non-typhoid *Salmonella* (NTS) infections occur in the United States each year (3). The frequent appearance of super bacteria and the breakthrough against the last line of defense, polymyxin, have also increased the risk of the global NTS infection crisis (4). Broad-spectrum antibiotics have the potential to disrupt the intestinal microbiota balance in treating enterobacterial gut infections because their antibacterial effects target both harmful and beneficial bacteria (5). Therefore, there is an urgent need for new antibacterial drugs that do not disrupt the balance of gut microbiota.

Microcins are a class of ribosomally assembled and posttranslationally modified peptides (RiPPs) produced under conditions when similar bacteria respond competitively, and they provide a competitive advantage in microbial communities (6, 7). Microcin-producing *Enterobacteriaceae* in human feces have been reported to contribute to gut microbial ecology. *E. coli* (8) (EcN), which produces microcins and limits the growth of competitors such as adherent invasive *Salmonella* in the inflamed intestine (8). However, EcN only reduces the *Salmonella* gut burden 17-fold and does not reduce inflammation (9). These results indicate that the strategy of microcin intervention to minimize the risk of *Salmonella* infection is feasible; more research is required to determine the optimal intervention and treatment strategies. Lasso peptides, one of the small molecules within microcins, have potential for application as next-generation drugs (10). The involvement of lasso peptide in the inflammatory competition among and within Enterobacteriaceae makes it a possible therapy for specifically inhibiting intestinal pathogens and limiting bacterial growth. Microcin J25 (MccJ25) administration to mice with prior enterostreptococcus infection resulted in reduced intestinal colonization by this pathogen, as well as limited growth of competing gut species, adherent invasive *E. coli* (11). MccJ25 protects against enterotoxigenic *Escherichia coli* (ETEC)-induced intestinal injury and inflammatory responses, suggesting the application of lasso peptide as an excellent antimicrobial or antiinflammation agent against pathogen infections.

A gene cluster isolated from *S*. Enterica has been demonstrated to encode microcin Y (MccY), a class I microcin that is 21 amino acids in length. Our previous study characterized the biological function of MccY as well as the basic chemical structure of the peptide and its potential mechanism of entry into cells to kill bacteria. RNA polymerase transcription inhibition using MccY against Enterobacteriaceae bacteria have been proved, and the bactericidal effect of MccY were observed at 0.05–0.8 µg/mL in *Shigella* and *Salmonella* strains (12, 13). While the role of MccJ25 as a factor influencing the host physiological development is established, NTS strains are highly resistant to MccJ25 due to specific receptor FhuA differences (10, 14). MccY exhibited the antimicrobial potency and structural stability in addressing NTS strains, and MccY is poised to function as an antibiotic alternative with applications in diverse fields ranging from antimicrobial agents and animal feed addition to the treatment of NTS bacterial diseases.

Evaluating the clinical application of MccY through *in vitro* trials presents apparent limitations, and the animal models are necessary to evaluate the impact of MccY on NTS-induced intestinal barrier protection. The research aimed to examine the impact of MccY on gut microbiota and intestinal barrier function by analyzing ecological shifts in murine intestinal physiology, systemic immunity, and microbiota. The study assessed the benefits and risks of *in vivo* MccY use to achieve a comprehensive understanding of MccY interactions in mice infected with NTS.

## RESULTS

### MccY has a high-intensity continuous bactericidal effect by inhibiting the activity of respiratory chain

**Fig 1:**
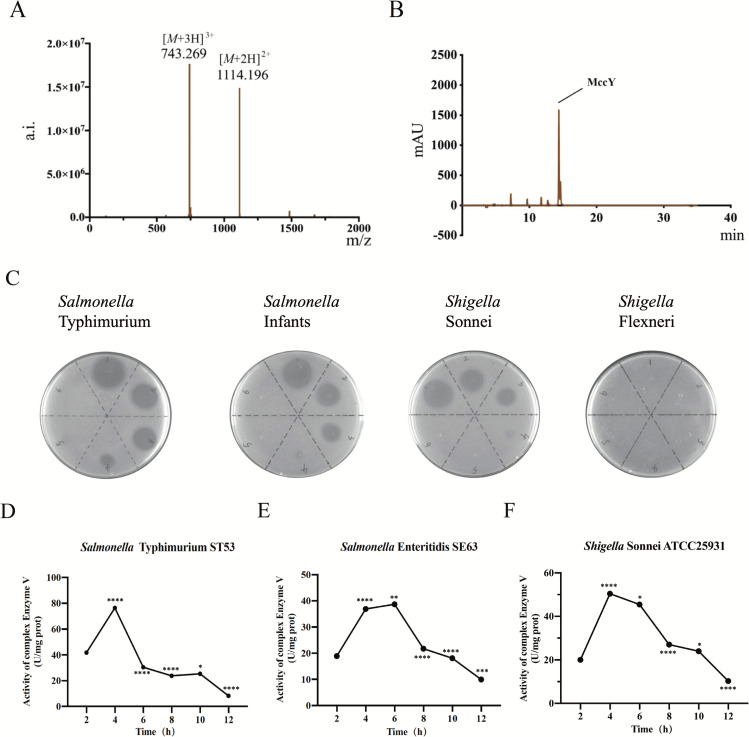
Preparation and characterization of MccY. (**A**) MccY identification was detected by LC–MS-QQQ-TOF. (**B**) MccY quantification performed by HPLC. The *x*-axis represents the charge-to-mass ratio, and the *y*-axis represents the response value. (**C**) The antimicrobial test of MccY against different serotype *Salmonella* and *Shigella* strains. (**D**). Activity of respiratory chain enzyme V of bacteria.

MccY identification and quantification were performed and described in Fig. 1A and B. Charged MccY species were detected at 1114.196 Da [M + 2 h]2+ and 743.269 Da [M + 3 h]3+. The absorption peak of MccY in the LC-MS-QQQ-TOF traces was observed at 14.52 min (280 nm). Fig. 1C showed the antimicrobial test of MccY proved that the inhibitory concentration of *S*. Typhimurium is 0.05 µg/mL, that of *S*. Infants and *S*. Sonnei is 0.10 µg/mL, and that of *S*. Flexneri was >1.0 µg/mL (Table S3). The results shown in Fig. 1D through F (Table S4) were analyzed in comparison to the previous time point. Strains could be killed by MccY inhibiting the activity of respiratory chain complex enzyme V within 12 h. In the logarithmic growth phase (4–6 h), ST53 and ATCC25931 enzyme were inhibited starting at 4 h (*P* < 0.0001); the SE63 strain was inhibited starting at 6 h (*P* < 0.0001). The results in the stationary phase (6–12 h) indicated that MccY inhibited the respiratory chain complex enzyme activities and the activity of ST53 (7.8 U/mg prot) was higher than those of SE63 (10.2 U/mg prot) and ATCC25931 (9.2 U/mg prot). It was confirmed that MccY has a high persistent bactericidal effect on foodborne bacteria by inhibiting the activity of respiratory chain complex enzyme V.

### Administration of MccY alleviated symptoms of infection and decreased the mortality rate and bacterial burden in infected mice

**Fig 2:**
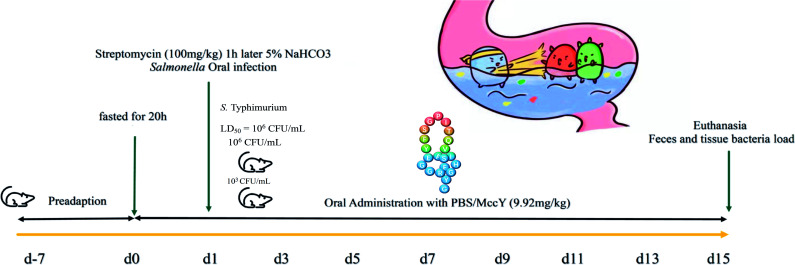
Experimental design for MccY for 2 weeks.

**Fig 3:**
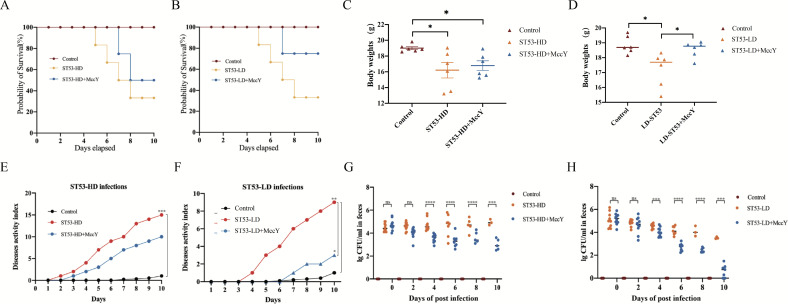
Therapeutic of MccY administration during *Salmonella* infection. (**A**) Percentage of survival of mice subjected to ST53-HD infection. (**B**) Percentage of survival of mice subjected to ST53-LD infection. (**C**) Body weight changes in ST53-HD-infected mice. (**D**) Body weight changes in the ST53-LD infection groups. (**E**) DAI in the ST53-HD infection groups. (**F**) DAI in the ST53-LD infection groups. (**G**) Detection of bacterial excretion in mouse feces in the ST53-HD infection and treatment groups. (**H**) Detection of bacterial excretion in mouse feces in the ST53-LD infection and treatment groups. Data are presented as the mean ± SD. Significant difference values are indicated for each group. (*t*-test; *P < 0.05, **P < 0.01, ***P < 0.001, and ****P < 0.0001).

The therapeutic effect of MccY on infections caused by foodborne opportunistic pathogens in the animal and human is still unclear. The experimental process in mice for the treatment of MccY after two different doses of infection was designed in Fig. 2, and the experimental process was based on previous studies of mouse models (11). The comprehensive detection method was referred to previous study on lasso peptide in mice, and the reference therapy dosage for MccJ25 in mice was 9.1 mg/kg body weight (BW) (15 – 17). In Fig. 3, the ST53-HD mice showed bloody stools on day 5 and succumbed to the infection on day 8. In contrast, the MccY treatment group showed delayed mortality for 1 day and a 20% increase in survival rate compared with the non-treated group (Fig. 3A). Similarly, the ST53-LD group mice died within 5–8 days, whereas those in the MccY treatment had a 40% increase (Fig. 3B). The average BW of ST53-HD mice was obviously decreased to the control group and the ST53-HD-MccY group (*P* < 0.05; *P* < 0.05) (Fig. 3C). The MccY-treated mice exhibited higher average BW compared with the ST53-LD mice (*P* < 0.05) (Fig. 3D). The weight loss observed in the infection group compared with the MccY-treated group indicates the effectiveness of MccY in preventing weight loss. The daily activity index (DAI) score (Table S5) was evaluated every day based on previous research on lasso peptide applications (11, 18). The overall assessment of DAI scores among the groups was provided in Fig. 3E and FTable S6 and S7. Infected mice displayed elevated body temperatures, ranging from 2°C to 5°C higher, and showed signs of bloody excretions. However, MccY treatment did not result in visible blood in the stool. The DAI score of ST53-HD groups has higher pathological score to the uninfected mice group (*P* < 0.001). On day 10, the pathological severity of MccY-treated mice was more severe (*P* < 0.01) than that of the uninfected group, but the score was lower than that of the infected group (*P* < 0.05). Thus, MccY alleviated the acute symptoms in the early phase of *Salmonella*-related bacteremia.

To investigate the effect of MccY on the clearance rate of *Salmonella*, the fecal bacterial count was based on the previous study on MccJ25 (18). On 3 dpi (Fig. 3G and H), a remarkable decrease in the levels of *Salmonella* was observed in the MccY-treated mice compared with the infection group. Mice inoculated with 10^6^ CFU/mL maintained approximately 10^4^ to 10^5^ CFU/mg *Salmonella*, and MccY reduced to 10^3^ CFU/mg feces, resulting in a sterilization rate close to 99%. Notably, the excretion status revealed a dramatic reduction in colonization load in the 10^3^ CFU/mL infection group to 10–20 CFU/mg, with a sterilization rate of 99.8%. Remarkably, MccY effectively eliminated a significant amount of *Salmonella* from the intestinal tract.

### MccY reduced the expression pro-inflammatory factors in intestinal mucosa

**Fig 4:**
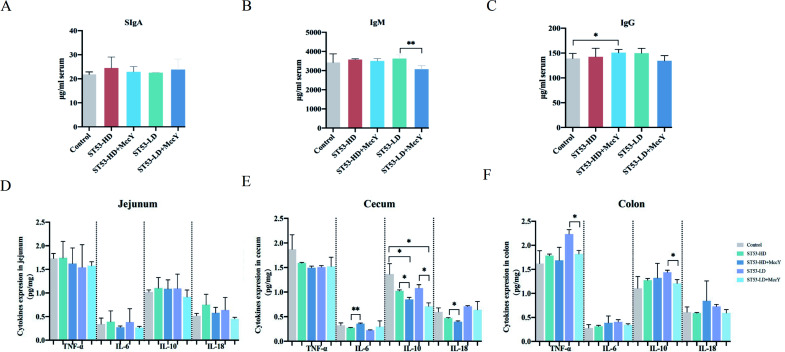
Changes in the expression of immune cytokines in serum in different groups. (**A**) SIgA expression of mice serum. (**B**) IgM expression of mice serum. (**C**) IgG expression of mice serum. (**D**) Cytokine expression in the jejunum. (**E**) Cytokine expression in the cecum. (**F**) Cytokine expression in the colon. Data are presented as mean ± SD; each group indicates significant difference values (*t*-test; *P < 0.05, **P < 0.01, ***P < 0.001, and ****P < 0.0001).

To explore the capacity of MccY to inhibit *Salmonella*-induced intestinal immunity, the levels of inflammatory cytokines were detected (Fig. 4). The serum of SIgA and IgM has no statistical changes, whereas the level of IgG in the ST53-HD-MccY group was increased (*P* < 0.05) relative to the control group (Fig. 4A through C). MccY decreased the level of IgM (*P* < 0.01) in contrast to the ST53-LD group, and SIgA and IgG shows no substantial difference. MccY showed a regulatory effect on the changes of IgM at the middle stage and IgG at the late stage, thereby modulating the humoral immune response and reducing the inflammatory response. NTS infection is associated with inflammatory bowel disease (IBD), which is characterized by chronic inflammation of the gastrointestinal tract. The main pathogenic of IBD cytokines involved in NTS inflammation includes TNF-α, IL-6, and IL-8 (19, 20). Intestinal secretion of TNF-α, IL-6, IL-10, and IL-18 was measured (Fig. 4D through F). No significant variations were observed among the different groups in the cytokine levels in the jejunum. However, IL-6 was appreciably increased in cecum in the ST53-HD-MccY group compared with the ST53-HD group (*P* < 0.01). The IL-10 in the cecum of infected mice was significantly decreased compared with the control group (*P* < 0.05), and further significant reduction was observed after MccY treatment. MccY treatment downregulated IL-18 in ST53-HD-challenged mice (*P* < 0.05); on the contrary, IL-6 was notably upregulated (*P* < 0.01). The changes in pro-inflammatory and anti-inflammatory factors in the colon were mainly exhibited in the ST53-LD-challenged mice. The expression of TNF-α (*P* < 0.05) and IL-10 (*P* < 0.05) decreased in the ST53-LD-MccY group compared with the ST53-LD-challenged group. MccY is involved in the regulation of intestinal mucosal immunity by reducing the secretion of pro-inflammatory factors, thereby enhancing the resistance of mice to NTS infection.

### MccY ameliorating intestinal villi shedding, dampening inflammation, and enhancing gut integrity

**Fig 5:**
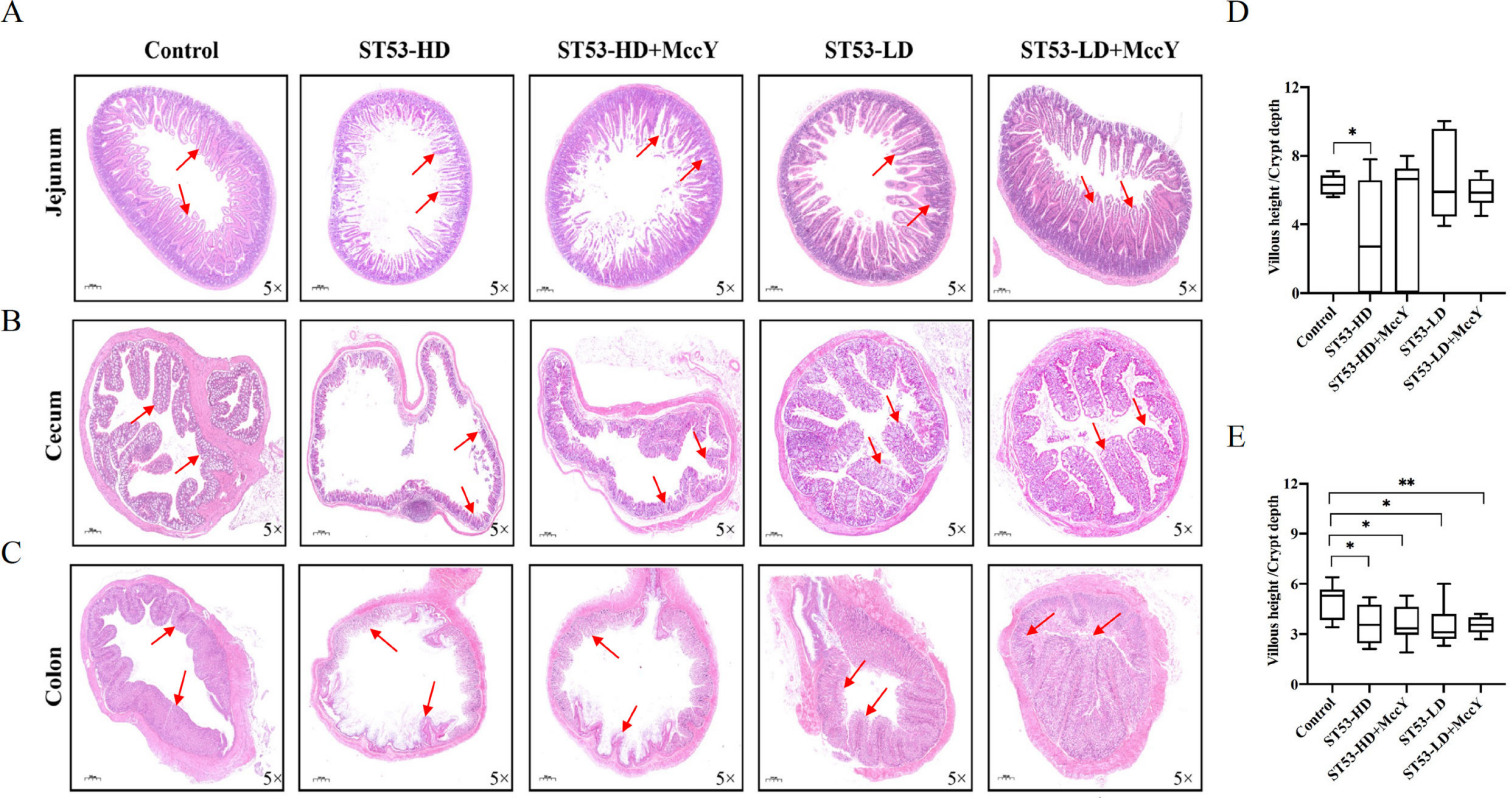
The effect of MccY on gut lumen morphology and inflammatory status in the ST53 infection BALB/c mice. Scale bar = 100 µm. (**A**) Images of the jejunum after H&E staining. (**B**) Images of the cecum lumen after H&E staining. (**C**) Images of the colon lumen after H&E staining. (**D**) Villous height and crypt depth ratio of the jejunum. (**E**) Villous height and crypt depth ratio of the colon. (Data were expressed as mean ± SD (*n* = 8), *t*-test; *P < 0.05, **P < 0.01, ***P < 0.001, and ****P < 0.0001 for MccY groups compared with control group).

Hematoxylin eosin (H&E) staining of the gut lumen further confirmed the intestinal protection of MccY during infection, and the histopathology and morphometrical characteristics were calculated in Fig. 5. The analysis excluded the data of cecum due to limited visibility of its crypt. In general, the intestinal damage degree of ST53-HD mice was significantly higher than that of ST53-LD mice. The jejunum of mice in the ST53-HD group exhibited severe intestinal tissue injury, including structural damage, and altered the functionality of cellular components such as goblet cells, Paneth cells, and neuroendocrine cells. The number of ST53-LD mice villi had been relatively maintained, and the mucosal morphology and crypt structure of jejunum in the MccY-treated group were protected (Fig. 5A). Specifically, in comparison to the jejunum, the cecum experienced less damage and a reduced shedding rate of goblet epithelial cells (Fig. 5B). In the ST53-HD group, the cecal mucosa and lamina propria were significantly separated, accompanied by villi shedding. However, in the ST53-LD infection group, the cecum exhibited a semi-exfoliative state, resulting in a monolayer of columnar epithelial cells. MccY-treated mice exhibited a general improvement in gut integrity, with minimal sloughing and an ordered arrangement of epithelial cells. After 14 days of infection, mice in the ST53-HD group displayed marked colitis (Fig. 5C), with almost complete loss and atrophy of the colon villi. The ST53-HD-MccY group demonstrated complete loss of most villi, and the colon showed a single-layered columnar epithelium with a thin brush border and a small number of scattered inflammatory cells within the lamina propria. The mucosal damage in ST53-LD mice was less severe compared with ST53-HD mice, and MccY therapy in mice colon resulted in no injury and did not exacerbate villus damage.

Inflammatory morphological changes in the intestine segment are indicative of chronic pathological changes in the gastrointestinal tract, and the villi and crypts are critical indicators of digestive and absorptive functions. Among the NTS-infected mice, several crypt epithelial cells were necrotic and sloughed off. The jejunum villi of ST53-HD mice were sparser, more damaged, and shorter, resulting in a lower V/C ratio in comparison with the control group (*P* < 0.05) (Fig. 5D). A common histological feature observed in colitis is the architectural distortion of the intestinal epithelium, characterized by the shortening and reduced branching of the crypts (21). MccY treatment led to alterations in the depth of colon crypts and a significant reduction in the colon V/C ratio compared with the control group (Fig. 5E). Based on these results, MccY therapy was found to decrease intestinal damage and enhance intestinal integrity.

### MccY promotes gut ecological balance by simultaneously reducing *Salmonella* burden

**Fig 6:**
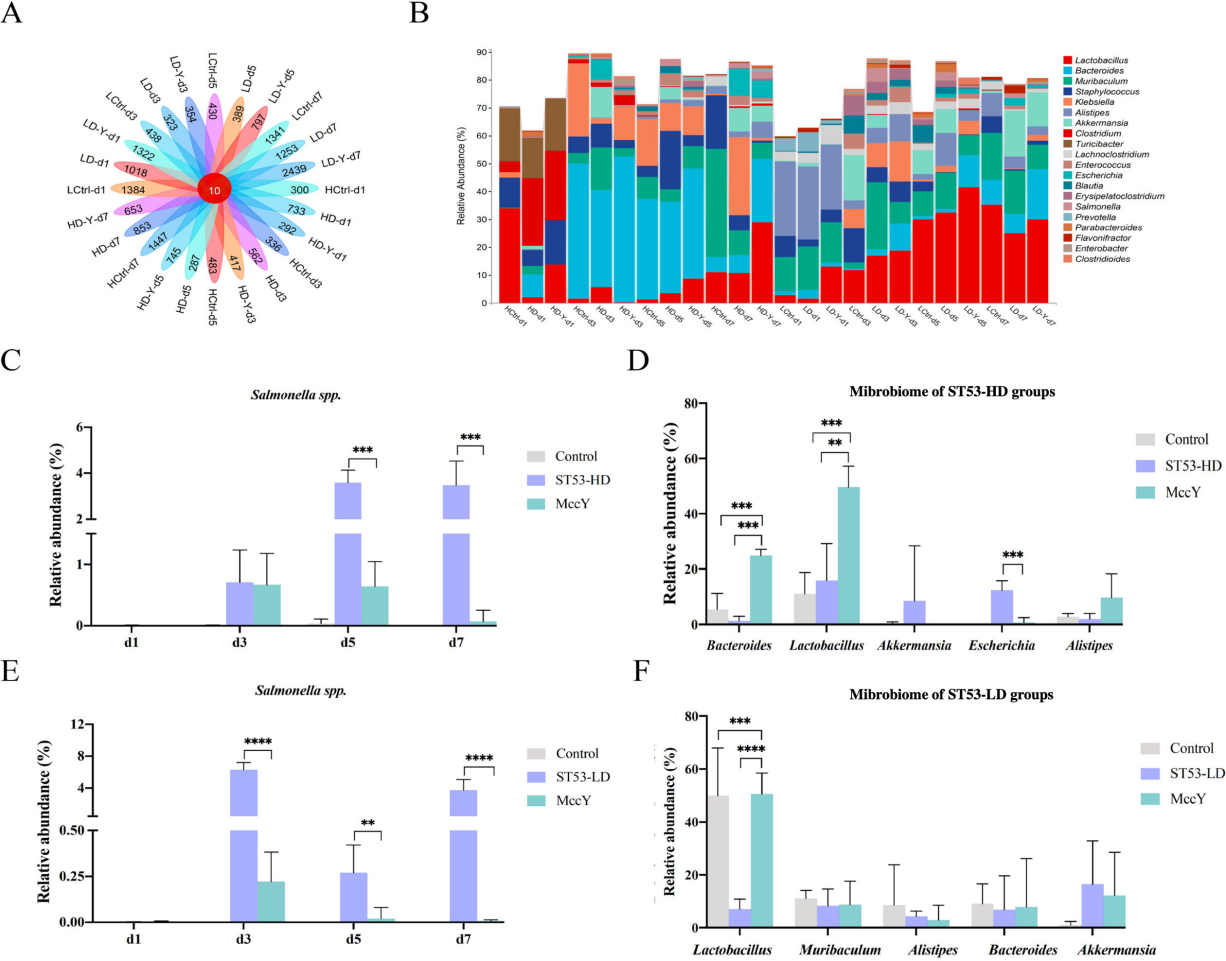
Effect of MccY treatment on the modulation of gut microbiota structure in the feces of the ST53 infection groups. (**A**) Venn diagram of ASV/OTU sequence abundance in different groups. (**B**) The relative abundance of fecal of mice. The *x*-axis represents each group of mice, and the *y*-axis represents the relative content ratio of each bacterial community to the total bacterial community at the genus level. (**C**) The relative abundance of *Salmonella* in the mouse feces in the ST53-HD infection groups. (**D**) The abundance of gut microbiota at the genus level post ST53-HD infection. (**E**) The relative abundance of *Salmonella* in the mouse fecal in the ST53-LD infection groups. (**F**) The abundance of gut microbiota at the genus level post ST53-HD infection. Each column indicates a group, and each row represents the genus level. Comparing MccY with controls, the data are presented as the mean ± SD. (*t*-test; *P < 0.05, **P < 0.01, ***P < 0.001, and ****P < 0.0001).

Mice feces were detected at four time points (1 day, 3 days, 5 days, and 7 days) to observe the dynamic changes of microflora (Fig. 6; Table S9 and S10 ). The number of amplicon sequence variation (ASV) in the samples varied substantially according to the Venn diagram (Fig. 6A). Specifically, the number of operations of the classification unit (OTU) decreased in the order of control group > ST53 MccY group > ST53 group. The Venn diagram indicated that MccY groups showed a greater microbial abundance than the infected group. The stacked bar charts in Fig. 6B aim to characterize the composition of various species in the microbiome. A higher ratio indicates a larger proportion of that particular bacteria in the total microbial flora. The community composition of the bacterial species in the ST53-HD group and ST53-LD group diverged significantly at the genus level. The main bacteria in the HD infection group were *Bacteroides* spp. and those of the LD infection group were *Lactobacillus* spp. A great proportion of *Salmonella* was observed in the ST53-HD-infected group, while the relative abundance of fecal *Salmonella* in mice administered with MccY was decreased on day 5 (*P* < 0.001) and day 7 (*P* < 0.001) (Fig. 6C). As described in Fig. 6D, the abundance of *Salmonella* in the ST53-LD-MccY-infected group was reduced than that in the MccY group (day 3, *P* < 0.0001; day 5, *P* < 0.01, and day 7, *P* < 0.0001). The MccY groups elucidated a dramatic reduction in *Salmonella* content, which was consistent with the results of *Salmonella* clearance depicted in Fig. 3G and H. MccY was more efficient at reducing the abundance of *Salmonella* in low-dose infections compared with high-dose infections.

Figure 6E and F analyzed the major flora abundance of each group in an average of 7 days. In the ST53-HD-infected mouse, the abundance of microbes decreased in the infected group; *Bacteroides* remained dominant after recovery from MccY treatment (*P* < 0.001). The abundance of *Bacteroides* was 19.7-fold higher in the MccY-treated groups than in the infection groups, and *Lactobacillus* increased significantly (*P* < 0.001). Interestingly, *Staphylococcus* and *Klebsiella* were significantly reduced (Fig. 6B), and the proportion of *Escherichia* that can be involved in intestinal bacterial infections was also inhibited, decreasing from 12.353% to 0.683% (*P* < 0.001) (Fig. 6E). *Lactobacillus* (50.602%) was the dominant species in the ST53-LD group, followed by *Bacteroides*, *Akkermansia*, *Escherichia*, and *Alistipes*. In Fig. 6F, the averages abundance of *Lactobacillus* in the treatment group was significantly higher compared with other groups (*P* < 0.001; *P* < 0.0001), accounting for 49.648% of the total microbial abundance in ST53-LD-MccY mice. The abundance of *Muribaculum*, *Bacteroides*, and *Akkermansia* did not markedly changes. MccY exerts multiple positive effects on gut microbiota ecology, including direct elimination of *Salmonella*, indirect inhibition of other opportunistic pathogens, and partial restoration of the microbiota.

### MccY constrains *Salmonella* transmission route, resulting in decreased burden in liver tissue

**Fig 7:**
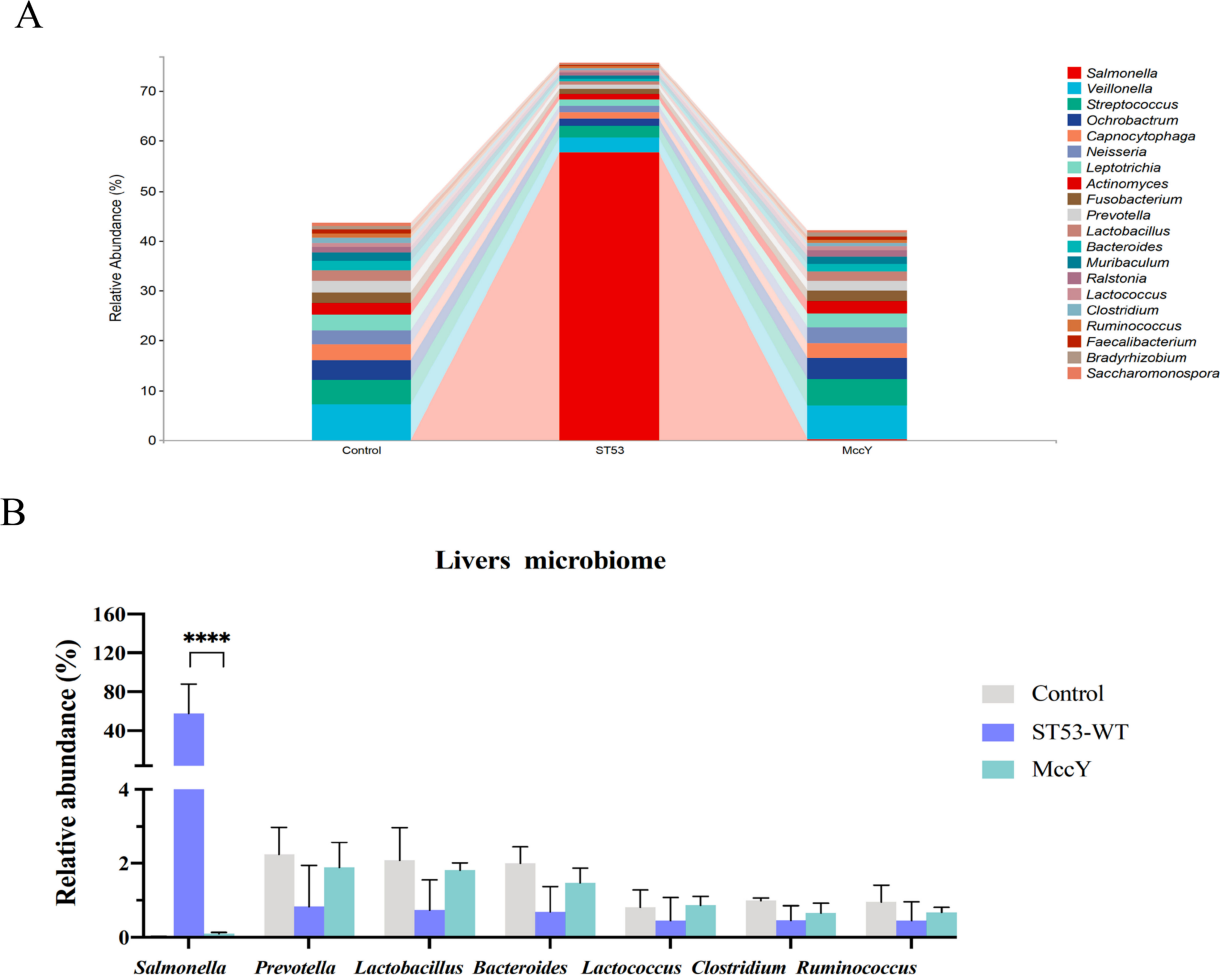
Effect of MccY on the modulation of liver microbiota structure of the ST53 infection groups. (**A**) The relative abundance of lives in the ST53 infection mice. (**B**) *Salmonella* composition in ST53 infection mouse livers. Each column indicates a group, and each row represents the genus level. Data are presented as the mean ± SD. Significantly different values are indicated for each group (*t*-test; *P < 0.05, **P < 0.01, ***P < 0.001, and ****P < 0.0001).

Further analysis of bacterial communities was conducted to investigate the mechanisms behind MccY supplementation enhancing host resistance against *S*. Typhimurium. The abundance of 20 major genus bacteria of ST53-LD group mice liver on day 14 was depicted in Fig. 7; Table S8. Liver samples from the control group and ST53-LD-MccY group have similar relative abundances with the main flora structure, primarily comprising of *Veillonella*, *Streptococcus*, *Ochrobactrum*, and *Capnocytophaga*. And the infection group of *Salmonella* content is the highest, followed by *Veillonella* and *Streptococcus*. Specifically, MccY decreased the ratio of *Salmonella* from the gut to liver, as compared with that of the infection group; *Salmonella* abundance of the MccY-treated group were dramatically decreased (*P* < 0.0001) (Fig. 7B). The concentration of several other bacteria, including *Prevotella*, *Lactobacillus*, *Bacteroides*, and *Lactococcus* increased, but no statistically significant differences were observed. MccY shows promise in limiting NTS infection to the gut level and curbing its dissemination to other tissues, thereby halting the *in vivo* transmission of NTS at its origin.

**Fig 8:**
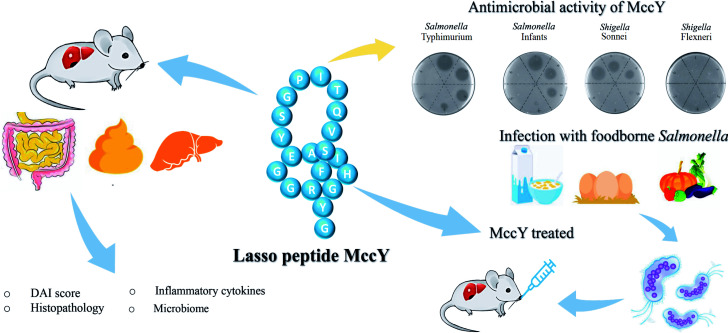
Biological function and application of MccY.

Consequently, the results demonstrated that MccY has the potential for therapeutic application in protecting against NTS *Salmonella-*induced intestinal damage and inflammation at different doses (10^6^ and 10^3^ CFU/mL). It also alleviates related intestinal dysbiosis and metabolic disorders (Fig. 8). This work provides into insights into the relationship between host and MccY, suggesting that MccY shows promise as a potential antimicrobial or anti-inflammatory agent for the treatment of pathogenic infections.

## DISCUSSION

In this study, the inhibitory activity of MccY against *Salmonella* was evaluated to demonstrate MccY bactericidal persistence *in vivo*. Mitochondrial respiratory complex V is known as F1F0-ATP synthase, which utilizes the energy produced by the gradient of protons across the membrane (22, 23). The sterilization data indicate that the efficacy of MccY persists, facilitating the targeted eradication of pathogens colonizing the digestive tract. In this work, mice challenged with *S*. Typhimurium from 1 dpi to 14 dpi displayed daily symptoms of inflammatory diarrhea and bloody diarrhea characteristic of bacillary dysentery. The results demonstrated that 9.92 mg/kg BW MccY prevented the onset of acute bowel disease in the early stage of infection, alleviated intestinal symptoms, and increased the survival rate by 20%–40%. The results in Fig. 4 presented that MccY upregulate the IgM level, and IgG results indicate that MccY play a crucial role in the immunity during the middle and late stages of infection.

The administration of MccY might contribute to higher levels of IL-6 in the cecum, particularly in cases of high-dose infection, through the expansion of intestinal epithelial cells. IL-6 maintains homeostasis by stimulating the proliferation and triggering an increase in neutrophil count, and IL-10 has been found to be negatively correlated with the occurrence and progression of gastrointestinal disease (24, 25). Bacterial metabolites regulate inflammatory body activation and IL-18 expression, ultimately influencing intestinal inflammation and barrier function (26). MccY maintain intestinal homeostasis by suppressing IL-10 and IL-18, mitigating damage to the mucous layer and inhibiting goblet cell maturation. MccY act as a mediator in intestinal inflammation by regulating TNF-α, IL-6, and IL-18, triggering an inflammatory response within the intestinal mucosa, leading to loss of integrity and increased permeability of the upper cortex. Villi are finger-like projections that protrude into the lumen of the intestine, increasing the surface area for nutrient absorption; crypts are formed by the intraepithelial lines surrounding the base of the villi (27). MccY contributed to adhesion of mucus and decreased epithelial adherence, inflammation, and desquamation, and the ratio of V/C decreased. MccY for therapeutic purposes did not exacerbate villus damage; it promoted gut integrity and mitigated overall intestinal damage yet.

MccY disrupted the early proliferation of *Salmonella* in the intestinal lumen, highlighting the targeted killing effect of MccY on enteric pathogens. The NTS strain remains dormant in the blood, waits for the right opportunity to replicate in the intestine, and becomes difficult to eliminate (28, 29). Intracellular *Salmonella* bacteria can be absorbed into the blood through the intestine and spread to the other tissue, such as the liver (30, 31). MccY has shown an inhibitory effect on the body, a small portion of *Salmonella* can enter the liver and replication, evading the effect of MccY. The similarity in the microflora distribution in the liver between the treatment group and the control group indicates the protective effect of MccY on mouse liver. MccY could confine NTS bacterial infection to the gut level and prevent its spread to other tissues, thereby preventing the transmission of *Salmonella*.

Microbial colonization is a major driver of changes in the gut metabolome (32, 33). MccY has a beneficial effect on the colonization and recovery of dominant bacteria. The ST53-HD-MccY and ST53-LD-MccY groups were primarily composed of *Bacteroides, Lactobacillus, Muribaculum*, and *Alistipes. Bacteroides*, as new-generation probiotics, inhibit pathogenic bacterial colonization and alleviate intestinal inflammation (34, 35). The abundance of *Lactobacillus* was notably increased, and previous research has reported that *Lactobacillus* reduces the susceptibility and severity of *Salmonella* infection, while *Alistipes* species produce short-chain fatty acids (SCFAs) and reduce intestinal inflammation (36, 37). These probiotics utilize MccY to compete with related species and colonize the gut, replacing the niche of intestinal pathogens. There was a markedly decreased abundance of *Staphylococcus* and *Klebsiella* in the ST53-HD infection group treated with MccY (Fig. 6B). MccY could reduce the proportions of opportunistic bacterial pathogen. The mode of action of MccY and its expected minimal impact on the microbiome will avoid the drawbacks of chronic antibiotic treatments. This study provides the first description of how MccY achieves potent therapeutic outcomes against NTS infection. MccY promoted a favorable gut microbial ecology and immune homeostasis. It effectively attenuates the elevated levels of *Salmonella* in the gut lumen and limits the expansion of opportunistic pathogen. This work demonstrated how microcin could be exploited to improve intestinal health and presents that the administration of MccY could be served as an alternative to antibiotic drugs.

## MATERIALS AND METHODS

### Bacterial strain and culture conditions

Primers and plasmid used in this study were designed and listed in Table S1. The *E. coli* and *Salmonella* strains used in this study were from our laboratory and are presented in Table S2. Briefly, strains were inoculated on 100 mL of LB solid medium and incubated overnight, 1:1,000 dilution in fresh LB broth. The broth was incubated for 6 h with 200 rpm shaking at 37℃. The samples were diluted 1:1,000 for the overlay tests.

### Preparation of microcin Y

The recombinant MccY plasmid was constructed in our laboratory (13). pYL01 was transferred into *E. coli* BL21 (DE3), then E. *coli* (YL02) was cultured in LB medium with 50 µg/mL kanamycin. The broth was induced with 0.5 mM IPTG and incubated for 24 h. The supernatant of the recombinant bacterial cell was collected by centrifugation. The purification and qualification of lasso peptide were performed using the QQQ-LC-MS approach, as described in our previous study. Inhibition assays on solid media were carried out with modifications to a previously published protocol with 0.005–1.0 μg/mL MccY. All experiments were independently repeated three times.

### Mitochondrial respiratory complex V activity assay

The mitochondrial respiratory complex V activity assay was conducted following the kit instructions. Bacteria in logarithmic phase were collected and diluted into 1 × 10^5^ CFU/mL and then treated with 0.2 mg/mL MccY culture for 2–12 h, and the bacteria were collected for each 2 h. The bacterial pellet was retained, and an appropriate amount of lysis buffer was added to the suspended cells and the mitochondria were extracted. The activities of mitochondrial respiratory chain complexes V were detected using a colorimetric method. The activity values of the complexes were calculated as follows:

Activity value = [(test sample OD value − background OD value) × system vol × test sample dilution multiple] / [test sample volume × X (millimole absorptivity) × reaction time (min) × test sample protein concentration (mg prot/mL)] Prot is an abbreviation of protein. The administered group samples were prepared in triplicate.

### Mouse experiments

All of the Specific Pathogen Free (SPF) mouse experiments were approved by the South China Agricultural University Institutional Animal Care and Use Committee, under ethics approval number 2021C084. Mice were purchased from Beijing Vital River Laboratory Animal Technology Co. Ltd. Six- to seven-week-old male and female SPF BALB/c mice were randomized and divided into six groups (control group, infected group, and treatment group, *n* = 10) and co-housed in groups of two to three under environmentally controlled conditions. Since the median lethal dose of ST53 is known to be LD_50_ = 10^6^ CFU/mL, two doses of infection were designed. The mice were infected with ST53 at two different doses: 10^6^ and 10^3^ CFU/mL. Before the infection, the mice were treated with 100 mg/kg BW streptomycin to rebuild their intestinal flora. One hour later, the animals were given 0.5% NaHCO_3_ to neutralize the stomach acid. MccY diluted in sterile PBS at 9.92 mg/kg was performed starting from 1 dpi and continued every day during the experimental period. Survival was monitored for 15 days, and exposure to CO_2_ is used to euthanize these mice.

### Disease activity index assessment

DAI assessment was performed by evaluating the physical signs and intestinal-related pathological indexes of mice before and during the experiments, as described previously (11, 38). Symptoms include body weight, body temperature, stool consistency, and presence of blood in the stool. Each mouse was scored for changes in body characteristics, and the scores for each group were added to obtain the average score. The DAI evaluation methods of three animal experiments were consistent.

### Cytokine production measurement

ELISA kits (Proteintech, USA) were used to measure serum SIgA, IgG, and IgM production. Blood samples were centrifuged at 2,000 *×* g for 10 min after collection. Total tissue protein was extracted by homogenization, and the concentration was measured using the BCA Protein Assay Kit (Novagen, Denmark). Plates were coated with a 5 µg/mL solution of carbonate buffer and incubated at 4°C for 1 h. Plates were washed and incubated for 1 h with peroxidase conjugated anti-HRP secondary antibody (Proteintech, USA) diluted 1:20 (vol/vol) with diluent. TMB Substrate Reagent Set was added and incubated for 30 min in the dark, and finally, 2N sulfuric acid was added to stop the reaction. Results were measured by absorbance at 450 nm, and ratios were calculated by dividing by the respective values. All assays were performed in duplicate, and a standard curve was generated for each experiment.

### Histological analyses

The gut lumen was removed from the mice and fixed in 40% paraformaldehyde, embedded in paraffin, and stained by hematoxylin eosin. Determination of the level of intestinal epithelial damage was based on the measurement of villus length and recess depth, followed by the use of software to calculate V/C. Changes in intestinal barrier integrity and inflammatory responses were assessed. The infection and pathology of mice after treatment were determined by assessing the presence of blood in the stool and degree of intestinal lesions after dissection I (9, 11).

### 16s rRNA gene sequencing and analysis

The microbiota analysis pipeline and bioinformatics analysis were performed according to previous reports. A total of 240 mouse fecal samples and 30 liver samples were collected, and DNA was extracted. The bacterial genome V3-V4 region of 16s rRNA was amplified by PCR (39). Amplicons were sequenced on an Illumina MiSeq platform using the MiSeq Reagent Kit v2 (500 cycles) (Personal Biotechnology Co. Ltd., China). Differences found in negative extraction controls prior to analysis were removed, after which the inverse Simpson diversity index and OTU richness were calculated using the unweighted UniFrac algorithm.

### Statistical analysis

GraphPad Prism 8 was used to calculate descriptive statistics, and all statistical comparisons of *P*-values were presented in the main and Additional files 1. One-way ANOVA analysis was used for comparisons of two and three or more groups. The results between each group were students *t*-tested to compare the significant differences between the groups. The data are presented as the mean ± SD.

## Data Availability

The authors confirm that the data supporting the findings of this study are available within the article and its supplementary materials. All data on microbial composition generated and/or analyzed during the current study are available in NCBI and the access number is PRJNA911349.

## SUPPLEMENTAL MATERIAL

Additional file1 spectrum.01784-23-s0001.xls Table S8 (Bacterial relative abundance data in of livers), Table S9 (Bacterial relative abundance data in the ST53-HD infection groups), and Table S10 (Bacterial relative abundance data in the ST53-LD infection groups).

Supplementary information spectrum.01784-23-s0002.pdf Table S1 (Primers and plasmids used in this study), Table S2 (Strains used in this study), Table S3 (The antimicrobial plate test results of MccY), Table S4 (The activity of respiratory chain complex enzyme V), Table S5 (Score of disease activity index [DAI] assessment), Table S6 (Scoring for calculating DAI of ST53-HD infection groups), and Table S7 (Scoring for calculating DAI of ST53-LD infection groups).

ASM does not own the copyrights to Supplemental Material that may be linked to, or accessed through, an article. The authors have granted ASM a non-exclusive, world-wide license to publish the Supplemental Material files. Please contact the corresponding author directly for reuse.
