# Supplementary material for: Lasso peptide MccY alleviates non-typhoidal salmonellae-induced mouse gut inflammation via regulation of intestinal barrier function and gut microbiota
Source: Microbiol Spectr. 2023 Oct 11;11(6):e01784-23. doi: 10.1128/spectrum.01784-23 (PMC10714986; doi:10.1128/spectrum.01784-23)
Supplement: Supplementary information — Table S1 (Primers and plasmids used in this study), Table S2 (Strains used in this study), Table S3 (The antimicrobial plate test results of MccY), Table S4 (The activity of respiratory chain complex enzyme V), Table S5 (Score of disease activity index [DAI] assessment), Table S6 (Scoring for calculating DAI of ST53-HD infection groups), and Table S7 (Scoring for calculating DAI of ST53-LD infection groups). [file spectrum.01784-23-s0002.pdf]

Supplementary information

Lasso peptide MccY alleviates Nontyphoidal Salmonellae - induced mice gut inflammation via regulation of intestinal barrier function and gut microbiota

Authors: Yu Li[1], Wenjing Li[1], Zhiwei Zeng[1], Yu Han[1], Qinxi Chen[1], Xinyi Dong[1], Zepeng Wang[1], Saixiang Feng[1,2,3,4,5*], Weisheng Cao[1,2,3,4,5*]

[1]College of Veterinary Medicine, South China Agricultural University, Guangzhou, China. [2]Key Laboratory of Zoonosis Prevention and Control of Guangdong Province, China. [3]Key Laboratory of Zoonosis of Ministry of Agriculture and Rural Affairs, Guangzhou, China. [4]Key Laboratory of Veterinary Vaccine Innovation of the Ministry of Agriculture and Rural Affairs, Guangzhou, China. [5]National and Regional Joint Engineering Laboratory for Medicament of Zoonosis Prevention and Control, China.

***Correspondence:**

Saixiang Feng: Tel/Fax, +86-20-85280718; E-mail: fengsx@scau.edu.cn

Weisheng Cao: Tel/Fax, +86-20- 85282536; E-mail: caoweish@scau.edu.cn

Table S1. Primers and plasmids used in this study.

Table S2. Strains used in this study.

Table S3. The antimicrobial plate test results of MccY.

Table S4. The activity of respiratory chain complex enzyme V.

Table S5. Score of disease activity index (DAI) assessment.

Table S6. Scoring for calculating DAI of ST53-HD infection groups.

Table S7. Scoring for calculating DAI of ST53-LD infection groups.

Table S8-S10 were offered in Additional file 1.

Table S8. Bacterial relative abundance data in of livers.

Table S9. Bacterial relative abundance data in the ST53-HD infection groups.

Table S10. Bacterial relative abundance data in the ST53-LD infection groups.

Supplementary Tables

Table S1. Primers and plasmids used in this study.

Primers	Sequence (5' to 3')	source
16s-338F	ACTCCTACGGGAGGCAGCA	
16s-806R	GGACTACHVGGGTWTCTAAT	
pYL01	*mcyABCD* gene cloned in pET28 (a) with BamHI and SalI, Kan^R	(1)

Table S2. Strains used in this study.

Strain	Relevant characteristic(s)	Source
YL01	pYL01 in *Escherichia coli* DH5α strain (F^-, φ80d/*lacZ*ΔM15, Δ(*lacZYA-argF*) U169 *recA*1 *endA*1 *hsdR*17)	(1)
YL02	pYL01 in *Escherichia coli* BL21 (F^-, *ompT* $hsdS_B$ (r_B^-, m_B^-) *gal dcm* (DE3))	(1)
ST53	*Salmonella* Typhimurium isolate ST53, wild type	(1)
SI52	*Salmonella* Infants, wild type	(1)
SE63	*Salmonella* Enteritidis, wild type	(1)
SS10	*Shigella* Sonnei, ATCC 25931	(1)
SS20	*Shigella* Flexneri, ATCC 29903	(1)

Table S3. The antimicrobial plate test results of MccY.

Strain	Relevant characteristic(s)	MIC (μg/mL)
ST53	*Salmonella* Typhimurium, wild type	0.05
SI52	*Salmonella* Infants, wild type	0.10
SS10	*Shigella* Sonnei, ATCC 25931	0.10
SF20	*Shigella* Flexneri, ATCC 29903	> 1.0

Table S4. The activity of respiratory chain complex enzyme V.

Complex enzyme V activity	2 h (U/mg prot)	4 h (U/mg prot)	6 h (U/mg prot)	8 h (U/mg prot)	10 h (U/mg prot)	12 h (U/mg prot)
ST53	41.51± 0.51	78.02 ± 0.50	34.49 ± 0.45	22.02 ± 0.45	23.53 ± 0.51	8.0 ± 0.48
SE63	19.50 ± 0.50	37.04 ± 0.21	38.54 ± 0.15	21.65 ± 0.10	19.09 ± 0.20	10.05 ± 0.50
SF20	20.01 ± 0.35	50.52 ± 0.50	47.45 ± 0.45	24.05 ± 0.55	22.55 ± 0.75	11.05 ± 0.53

Table S5. Score of disease activity index (DAI) assessment.

Score	Weight loss	Temperature	Stool consistency	Blood
0	None	Normal	Normal	Negative hemoccult
1	1-5 %	± 0.5 ℃	Soft but still formed	Negative hemoccult
2	6-10 %	± 1.0 ℃	Soft	Positive hemoccult
3	11-18 %	± 1.5 ℃	Wet and very soft	Blood traces in stool visible
4	18 %	± 2.0 ℃	Watery diarrhea	Gross rectal bleeding
5	death			

The DAI scoring rules refer to the previous experimental experience (2, 3).

Table S6. Scoring for calculating DAI of ST53-HD infection groups.

Days	Control	ST53-HD	ST53-HD-MccY
1	0.001 ± 0.0001	0.005 ± 0.003	0.0001 ± 0.01
2	0.001 ± 0.022	1.097 ± 0.010	0 .0013 ± 0.01
3	0 .001 ± 0.002	2.047 ± 0.0202	1.053 ± 0.025
4	0.002 ± 0.001	4.001 ± 0.0026	2.064 ± 0.073
5	0.002 ± 0.003	7.201 ± 0.00002	3.148 ± 0.042
6	0.002 ± 0.023	9.023 ± 0.0003	5.216 ± 0.062
7	0.202 ± 0.002	10.02 ± 0.246	7.052 ± 0.018
8	0.301 ± 0.015	13.12 ± 0.07	8.006 ± 0.018
9	0.507 ± 0.012	14.03 ± 0.0007	9.185 ± 0.009
10	1.307 ± 0.024	15.13± 0.023	10.095 ± 0.0212

Table S7. Scoring for calculating DAI of ST53-LD infection groups.

Days	Control	ST53-LD	ST53-LD-MccY
1	0 .001 ± 0.008	0.001 ± 0.001	0.00 ± 0.0001
2	0.001 ± 0.002	0.001 ± 0.0002	0 .00 ± 0.0016
3	0 .002 ± 0.003	0.021 ± 0.001	0 .00 ± 0.022
4	0 .006 ± 0.015	1.01 ± 0.002	0.0001 ± 0.032
5	0.016 ± 0.026	3.14 ± 0.002	0 .0001± 0.011
6	0.128 ± 0.015	4.05 ± 0.006	0.001 ± 0.042
7	0.262 ± 0.014	6.01 ± 0.064	1.01 ± 0.007
8	0.345 ± 0.054	7.02 ± 0.052	2.37 ± 0.003
9	0.401 ± 0.042	8.06 ± 0.074	2.39 ± 0.003
10	0.935 ± 0.002	9.13 ± 0.002	3.01 ± 0.002

References

1. Li Y, Han Y, Zeng Z, Li W, Feng S, Cao W. 2021. Discovery and Bioactivity of the Novel Lasso Peptide Microcin Y. J Agric Food Chem 69:8758-8767.
2. Shang L, Yu H, Liu H, Chen M, Qiao S. 2021. Recombinant antimicrobial peptide microcin J25 alleviates DSS-induced colitis via regulating intestinal barrier function and modifying gut microbiota. Biomedicine & Pharmacotherapy 139:111127.
3. Sasaki M, Mathis JM, Jennings MH, Jordan P, Wang Y, Ando T, Joh T, Alexander JS. 2005. Reversal of experimental colitis disease activity in mice following administration of an adenoviral IL-10 vector. Journal of Inflammation 2:1-10.
